# Multi-omics integration uncovers epigenetic control of metabolic reprogramming in triple-negative breast cancer

**DOI:** 10.1186/s43046-026-00401-7

**Published:** 2026-09-02

**Authors:** Esraa M Hashem, Mai S. Mabrouk

**Affiliations:** 1 Misr University for Science and Technology, Cairo, Egypt; 2 Center for Informatics Science (CIS), School of Information Technology and Computer Science, Nile University, Cairo, Egypt

**Keywords:** Triple-negative breast cancer (TNBC), DNA methylation, Metabolic reprogramming, Differential gene expression, Integrative omics, Epigenetic dysregulation, Survival analysis

## Abstract

Triple-negative breast cancer (TNBC) is an aggressive subtype characterized by the absence of estrogen, progesterone, and HER2 receptors, limiting effective targeted therapies. Increasing evidence suggests that metabolic reprogramming, a hallmark of TNBC progression, is driven by underlying epigenetic mechanisms such as DNA methylation. The represented study performed an integrative analysis of transcriptomic (RNA-seq) and methylome data to uncover the metabolic–epigenetic interplay in TNBC. Differential gene expression analysis using DESeq2 revealed significant dysregulation of key metabolic genes, including upregulation of genes encoding glycolytic and serine biosynthesis enzymes and downregulation of metabolic tumor suppressors. Genome-wide methylation profiling identified extensive cytosine–phosphate–guanine (CpG) hypermethylation events associated with transcriptional repression, particularly in promoter regions. Integrative analysis pinpointed a subset of metabolism-related genes exhibiting both differential expression and methylation, such as *FBP1*, *RASSF1A*, and *PHGDH*. Pathway enrichment analysis highlighted aberrations in glycolysis/gluconeogenesis, fatty acid metabolism, and one-carbon pathways (adjusted *p* < 0.01). Importantly, TNBC patients with hypermethylated metabolic gene signatures displayed significantly shorter overall survival (log-rank *p* < 0.05). These findings reveal that DNA methylation-driven metabolic dysregulation contributes to TNBC aggressiveness and may provide novel biomarkers and therapeutic targets at the metabolic–epigenetic interface.

## Introduction

Breast cancer is the most frequent malignancy and the leading cause of cancer death among women globally, with 2.3 million new cases and 685,000 deaths in 2020, according to GLOBOCAN [1]. Its incidence is increasing worldwide, and although there have been significant advances in both early detection and therapeutic strategies, 5-year survival rates differ greatly among disease subtypes, geographical locations, and access to care [2]. According to pathological and biomarker features, breast cancer is currently classified into three major clinical subtypes: hormone receptor (HR) positive, HER2-positive BC, and TNBC, in the treatment and prognostic settings [3]. Among these subtypes, TNBC represents 10–15% of all breast cancers, but it is the deadliest subtype that is at high risk of recurrence and early metastasis, and poor overall survival, mainly because of a lack of expression of estrogen receptor (ER), progesterone receptor (PR), or human epidermal growth factor receptor 2 (HER2) [4].

TNBC cancers constitute a group of clinically and genomically heterogeneous tumors, displaying molecular heterogeneity, metabolic heterogeneity, and histological heterogeneity, making diagnosis, prognosis, and treatment extremely complex [5]. This heterogeneity highlights an immediate requirement for new biomarkers and targets for therapy, which are more specific to TNBC biology. As in most tumors, breast cancer progression is strongly associated with metabolic reprogramming. Cancer metabolism, a product of energy reprogramming, is unique and established as one of cancer’s hallmarks [6]. To cope with the higher need for energy, biosynthetic precursors, and redox balance of cancer cells, these latter reprogram their metabolic activities [7]. Thus, substantial remodeling of cancer cell metabolism is required not only for producing anabolic macromolecules in order to fuel the growth of cancers such as breast tumors, but also to sustain a transformed state and support cellular proliferation [8].

In addition, intrinsic genetic and epigenetic changes, such as oncogene activation and tumor suppressor inactivation, also contribute to these metabolic alterations [9] One such topic of growing interest is the relationship between cell metabolism and epigenetic control, in particular, DNA methylation [6]. Tumor cells often reprogram their metabolism and epigenetic profiles to support rapid proliferation and therapeutic resistance.

DNA methylation, that is catalyzed by DNA methyltransferases (DNMTs), consists of the transfer of a methyl group to cytosine residues at CpG dinucleotides and generally correlates with silencing the transcription of tumor suppressor genes [10]. DNA methylation is catalyzed by DNA methyltransferases as a group of enzymes, which include DNMT1 (DNA (cytosine-5)-methyltransferase 1), DNMT3A, and DNMT3B. They have a similar structure with large N-terminal regulatory and C-terminal catalytic domains but play quite different roles in vivo; they also exhibit specific tissue expression patterns [11]. Conversely, DNA demethylation processes are also capable of activating oncogenic pathways, promoting tumor heterogeneity and neoplastic progression [12]. In TNBC, methylation characteristics are similar to other breast cancer subtypes including global genomic hypomethylation and regional hypermethylation at CpG islands; however, the specific genes methylated, and the extent of their alteration vary significantly among subtypes [13]. The main target sites for DNA methylation are cytosine–phosphate–guanine dinucleotides (CpG, where a C and G nucleotide follow each other) [14].

It has been estimated that about 70% of human promoters are CpG islands, whose methylation is closely correlated with transcriptional activity [15]. Abnormal methylation of those promoters induces a disturbance in the expression of some-related genes and is responsible for the aggressive clinical behavior of TN tumors.

Emerging evidence expanding recent studies has suggested that epigenetic reprogramming and metabolic remodeling are functionally intertwined in breast cancer. For instance, Dong et al. (2013) have revealed that the gluconeogenic enzyme FBP1, a significant glycolysis repressor, is under an epigenetic silencing condition through Snail DNMT1-induced promoter hypermethylation (HPM) of its promoter in basal-like and TNBC (triple-negative breast cancer) cells, resulting in induced glycolytic flow and augmenting tumor invasion [16]. Gong et al. (2021) applied integrative omics for the classification of TNBC into three metabolic subtypes (glycolytic, lipogenic, and oxidative), which exhibit unique clinical outcomes and therapeutic susceptibilities [17]. These results indicate direct prognostic implications of metabolic heterogeneity in TNBC.

In addition to these observational profiling, mechanistic studies have indicated that metabolic intermediates and epigenetic enzymes are causally responsible for the scene. For instance, S Friso (2017) indicates that variations in the levels of methionine and its metabolite, S-adenosylmethionine (SAM), can influence global methylation patterns and gene expression in breast cancer cells, providing direct biochemical evidence for a connection between one-carbon metabolism and DNA/histone methylation [18]. Moreover, this association is reinforced by the fact that overexpression of enzymes such as MAT2A and SHMT2, which are involved in SAM production and serine-glycine metabolism, promote hypermethylation and tumor progression in TNBC [19]. Liu et al. (2016). Further, among the hypermethylated genes is also ENPP2 (autotaxin), a lipid-metabolism enzyme that has been included in a diagnostic methylation panel for discriminating TNBC from all other BC subtypes [20].

All together, these results highlight the crosstalk between metabolism and epigenetics in both directions: metabolites serve as cofactors for DNA- and histone-modifying enzymes, whereas epigenetic modifications regulate metabolic gene expression. Nevertheless, even with such progress, there is still a dearth of integrated studies that comprehensively correlate metabolic gene mRNA levels with DNA methylation alterations in TNBC. To bridge this gap, in the present study, we utilized a combined analysis of transcriptomic and DNA methylation profiling of TNBCs and paired non-tumor adjacent breast tissues to discover the metabolically relevant genes under epigenetic regulation. Through integration of differentially expressed and methylated signatures, as well as pathway enrichment and survival modeling, the study was designed to identify epigenetically perturbed metabolic pathways driving TNBC development and prognosis. A better understanding of this metabolic–epigenetic crosstalk could unveil new aspects of TNBC biology and, ultimately, novel biomarkers or druggable epigenetic targets on the crossroad between metabolism and epigenetic regulation.

## Materials and methods

**Fig. 1:**
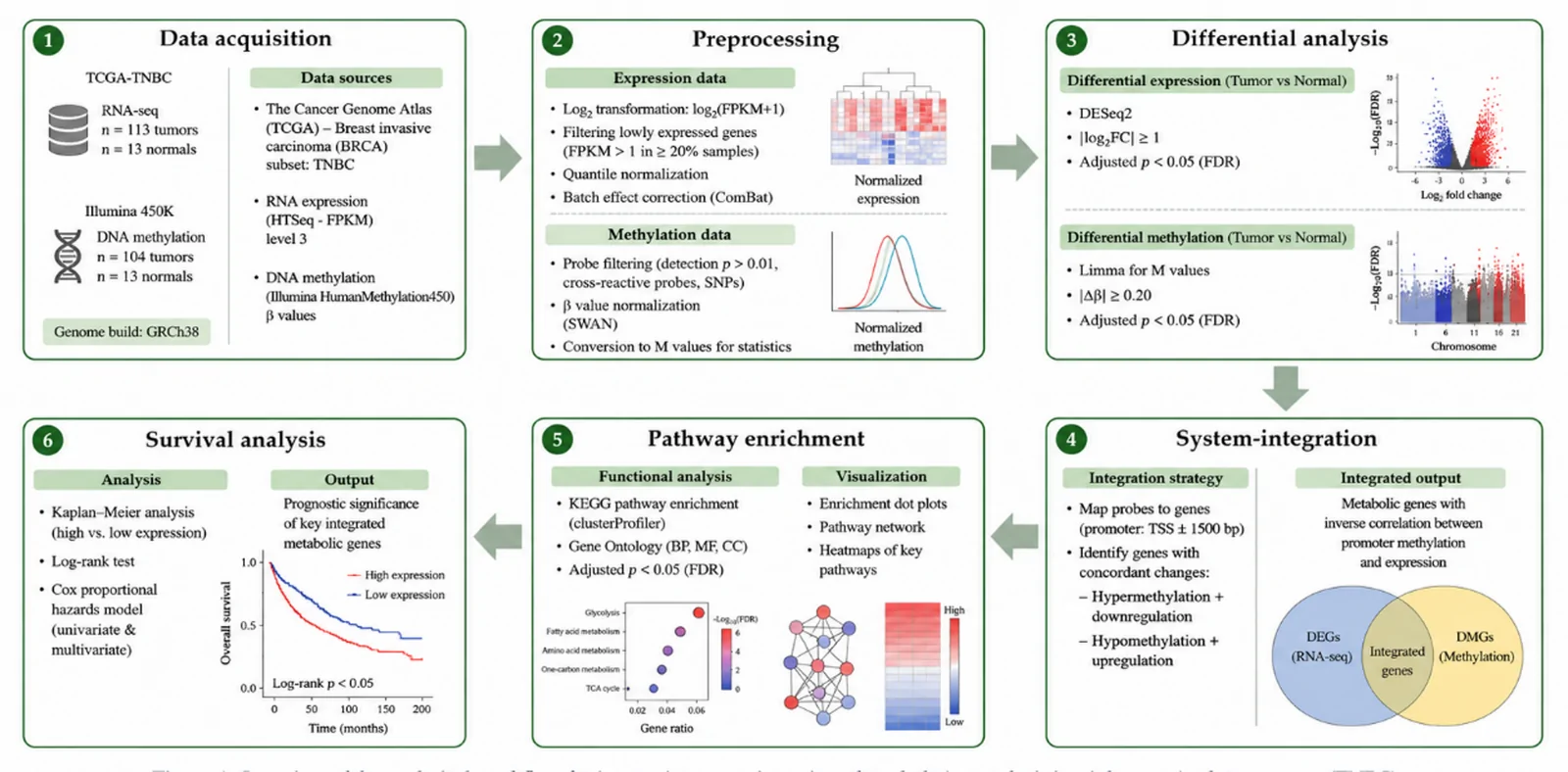
Overview of the analytical workflow for integrative transcriptomic and methylation analysis in triple-negative breast cancer (TNBC)

In order to clarify the metabolic remodeling in relation to DNA methylation alterations in TNBC, this study relied on a multistep integrative analysis, as illustrated in Fig. 1. TNBC samples were defined from the TCGA-BRCA cohort based on clinical annotations indicating negative status for estrogen receptor (ER), progesterone receptor (PR), and HER2. Only primary tumor samples with complete RNA-seq and DNA methylation data were included.

The workflow started with downloading available public transcriptomic and methylation data sets of TNBC and normal breast tissue samples. Patients were stratified into high-risk and low-risk groups based on the median value of the metabolic–epigenetic score. At the later phase, the differentially expressed genes (DEGs) from RNA-seq data and differentially methylated regions (DMRs) from methylation arrays were identified following intensive data preprocessing and quality control. The two datasets were combined for the identification of the metabolic genes showing an inverse association between methylation level and gene expression level as candidates for epigenetic dysfunction. Functional annotation was conducted by KEGG and Reactive pathway enrichment. Finally, the clinical relevance of these genes was validated by correlation and survival analyses. This integrative approach offers a holistic systems-level view of the metabolic–epigenetic interplay contributing to TNBC pathogenesis and highlights candidate biomarkers for targeted intervention.

### Data acquisition and preprocessing

**Fig. 2:**
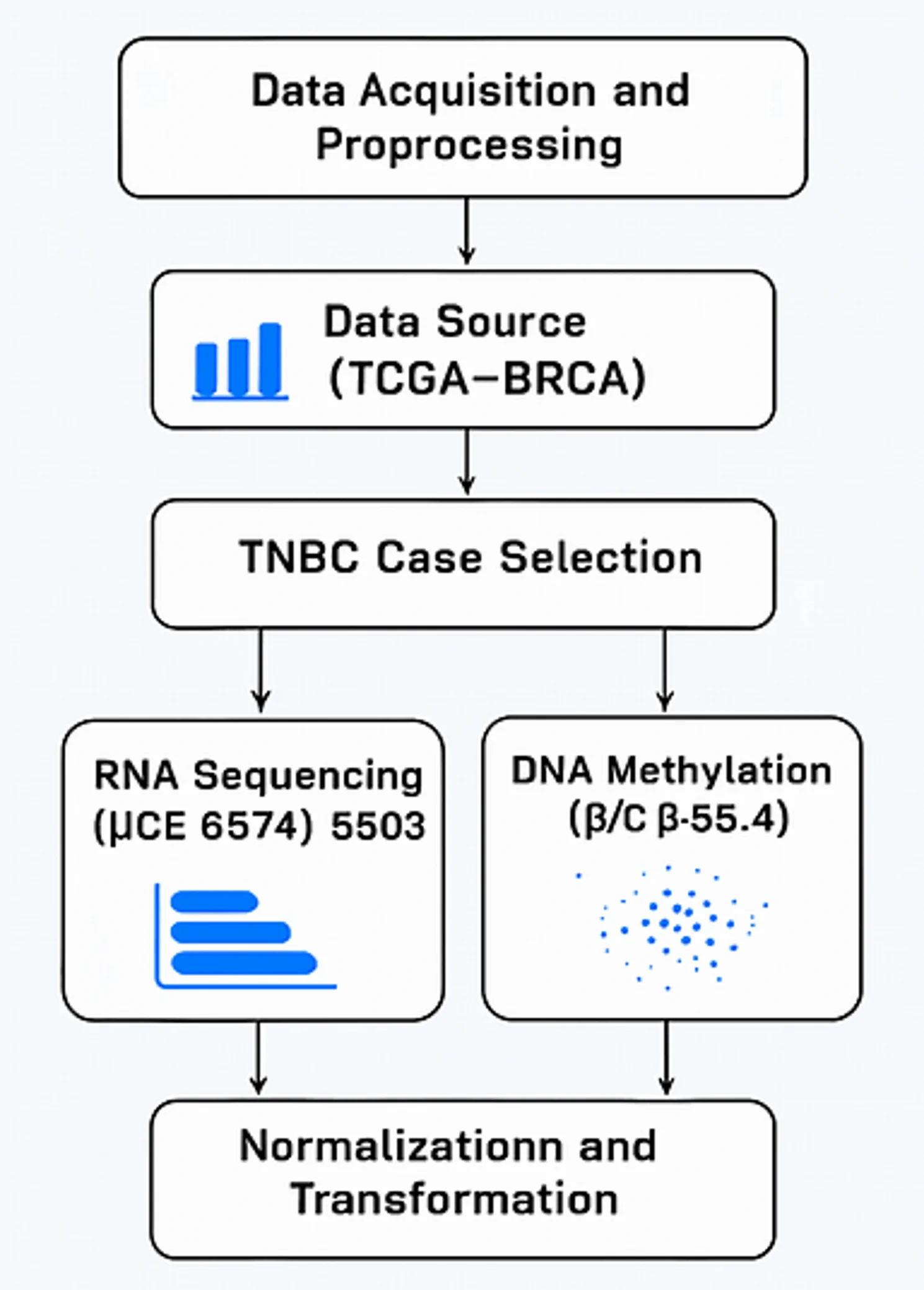
Data acquisition and preprocessing steps for TNBC

Utilizing publicly available multi-omics data for RNA-seq and DNA methylation (Illumina HumanMethylation450) data for breast cancer were obtained from the Cancer Genome Atlas (project ID: TCGA-BRCA) via the Genomic Data Commons (GDC) Data Portal [21]. Figure 2 describes the data acquisition and preprocessing pipeline.

TNBC case selection: The cases of breast cancer that were triple negative (ER-negative, PR-negative, HER2-negative), subtype were selected from the clinical metadata of TCGA. In total, *N* ≈ 120 tumors from patients with TNBC (according to the TCGA information on the status of the receptors) were processed for analysis. As controls, we selected *N* ≈ 100 normal breast samples from TCGA. Numbers of mapped reads were normalized and transformed (using DESeq2 variance-stabilizing transformation) to control for library size, Table 1 [22]. The normal samples were primarily solid tissue normal controls and were not fully matched to tumor samples on a per-patient basis.

**Table 1:** Characteristics of the TCGA TNBC Dataset

Data Type	TNBC Tumor Samples	Normal Samples	Median Age (Years)	Stage Distribution	Platform	Description
RNA-Seq (Gene Expression TCGA-BRCA)	120	100	55	I–IV	Illumina HiSeq 2000	Raw read counts normalized with DESeq2
DNA Methylation (450 K Array (Illumina HumanMethylation450 BeadChip)	115	98	54	I–III	Illumina HumanMethylation450	β-values normalized with BMIQ
Clinical Data	120	100	–	–	TCGA Metadata	Includes receptor status, survival time, and tumor size

Methylomic data TCGA 450 K array data were employed to analyze DNA methylation. Methylation β-value (0–1) of each CpG probe was extracted, and probes containing SNPs or poor signals were removed. Methylation data were intra-sample normalized and batch corrected using the ChAMP BioConductor pipeline [22]. Following preprocessing, we selected methylation data for 450,000 CpG sites and expression data for 20,000 genes in each sample. All data analysis was performed using R with packages (TCGA biolinks, DESeq2, ChAMP/minfi) and custom Python scripts for data manipulation. Differential expression analysis was performed using DESeq2, a widely validated method that remains a gold standard for RNA-seq analysis due to its robust normalization and dispersion estimation. Although established, such methods ensure reproducibility and comparability with prior large-scale studies, including TCGA-based analyses.

### Differential gene expression analysis

Transcriptional changes between TNBC and normal breast tissue were indicated by DGE analysis. For differential analysis of count data, we utilized shrinkage estimation for dispersions and fold changes to make estimates more stable and interpretable, implemented in the form of the DESeq2 package [23] a statistical framework that is broadly used for analyzing RNA-seq data due to its flexibility and robustness in modeling count-based data. DESeq2 uses the negative binomial distribution to model gene-level count data, which enables estimation of mean and variance accurately, as well as the consideration of biological and technical variability.

Genes with very low reads were removed before analysis in order to reduce background noise and increase the power of detection. Subsequently, the filtered dataset was normalized using the median-of-ratios approach to adjust for sequencing depth and compositional effects across samples, which incorporates biological differences rather than technical variation into downstream comparison [23].

**Fig. 3:**
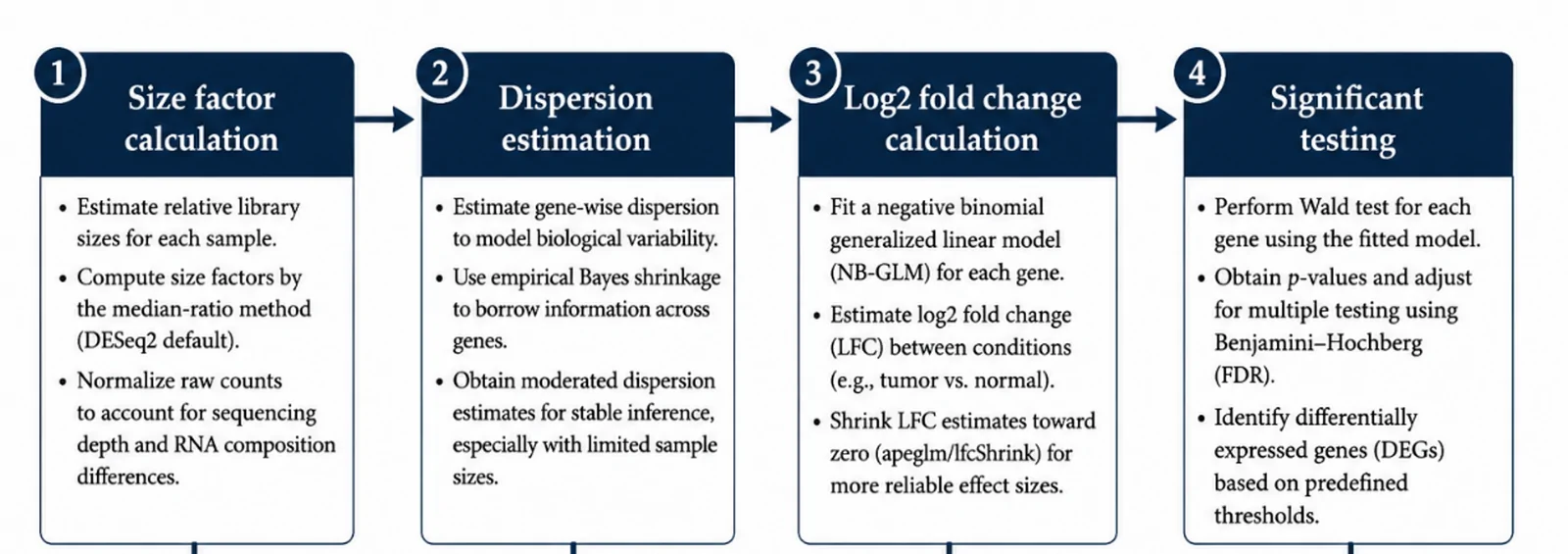
DESeq2 workflow. To identify genes that are transcriptionally altered in TNBC, we performed differential gene expression analysis using DESeq2, comparing TNBC tumor samples to normal breast tissues. A total of *X* genes was found to be significantly dysregulated (FDR < 0.05, |log₂FC| ≥ 1)

The DESeq2 pipeline consisted of three major steps, summarized in Fig. 3: (1) estimation of size factors for normalization across libraries; (2) fitting a single dispersion parameter using an empirical Bayes approach to borrow information across genes to achieve more stable estimates of variance; and (3) estimating log 2-fold changes from a generalized linear model. For significance testing of the association between FEC and lymphocyte expression levels, and the correlation among clonal expansion scores and peripheral immunophenotypes the Spearman (non-parametrical) test was used.

### Differential DNA methylation analysis

DNA methylation is an important epigenetic modification involved in the expression of genes and chromatin structure [24]. Therefore, we also performed a genome-wide differential methylation analysis to identify CpG sites and regions with DNA methylation significantly different in TNBC tumors compared with normal tissue using the combined R packages ChAMP pipeline and limma linear models [25, 26], that package data preprocessing and analyzing tools, then used limma linear models [27] to assess the significance of each individual probe’s differential methylation between groups (TNBC vs. normal). P for all analyses: adjusted for multiple comparisons procedures with Benjamini-Hochberg false discovery rate (FDR) correction [28].

Hypermethylated probes in TNBC were defined as those for which the mean β-value (percentage of methylated cytosines at each CpG site) was markedly increased in tumors compared to matched normals with a false-discovery rate (FDR) < 0.05 and Δβ ≥ 0.2. Alternatively, probes were deemed hypomethylated if they had an inverse β-value in tumors versus normal (tumor β-value < normal) with FDR < 0.05 and Δβ ≤ −0.2. This thresholding yielded thousands of differentially methylated CpGs throughout the genome.

To avoid spurious findings, we generated differentially methylated regions (DMRs), by taking into account the surrounding CpG probes that have also passed the significance threshold simultaneously by using DMRcate [29], to identify hotspots in methylation showing biological relevance. A large proportion of the DMRs were located at gene promoter regions (± 1 kb around the transcription start site (TSS) of genes). It is well established that promoter hypermethylation correlates with gene silencing, which is a signature mark of cGAS epigenetic dysregulation in tumorigenesis [30].

Consistent with prior breast cancer methylation studies, we found that promoter hypermethylation events were most prevalent in SBR, as compared to normal tissues, and this phenomenon leads us to infer the silencing of tumor suppressor genes. It was observed indeed that a proportion of the hypermethylated loci are mapped to well-established TSGs included in our reference list, such as RASSF1A and CDKN2A/p16 [31]. Furthermore, we observed global hypomethylation in intergenic regions and repetitive elements, which is a common property of cancer-related methylomes.

### Batch effect correction

To minimize technical heterogeneity between samples, batch effects were evaluated and adjusted before downstream analyses. Normalization and batch correction were done using [e.g., ComBat from the sva package/limma normalization methods] for RNA-seq and DNA methylation data. This provided comparability across samples and reduced non-biological variation.

### Integrative identification of epigenetically dysregulated metabolic genes

Most gene expression profiling studies in TNBC have been performed to identify better prognostic or predictive markers; we integrated both the RNA-seq and methylation data to find genes that are dysregulated epigenetically across TNBC. performed by correlating promoter methylation levels (Δβ values) with corresponding gene expression levels. Genes showing significant inverse correlation (hypermethylation with downregulation or hypomethylation with upregulation) were defined as methylation-driven genes, using a defined list of candidate genes based on (1) up-regulated/down-regulated gene expression in TNBC compared to normal (DEG), and (2) presence of a significant DMR in the gene promoter region when comparing the same two sample groups. Out of the ~ 20k genes [32], this integrative filter gave rise to X genes integrated expression and methylation changes. We analyzed the direction of change for each gene to draw conclusions about regulatory relationships. In a large number of instances, we found an inverse correlation: promoter hypermethylation was found to be associated with gene downregulation (presumed transcriptional silencing by methylation), and the opposite for hypomethylation. This is not surprising since DNA methylation in promoters has been shown to repress gene transcription [33]. We verified this inverse correlation in the 450 K array by plotting gene expression versus promoter β-values for selected genes across samples. Importantly, we overlapped the candidate list with known metabolic genes to highlight metabolic-epigenetic crosstalk. 1027 Metabolic genes were 1028 defined via gene annotation according to KEGG and Gene Ontology (GO) Biological Process category. Some Y of the X epigenetically modulated genes were involved in metabolism. These are metabolically relevant, epigenetically dysregulated genes, and the major discovery of the presented study. To further limit the confidence in these findings without experimental validation, we performed multi-level in silico validation: (i) concordance analysis between DNA methylation and gene expression data; (ii) pathway enrichment consistency for found genes; or (iii) survival analysis to verify clinical relevance.

They consist of central metabolic and biosynthetic genes, such as those involved in central carbon metabolism, lipid metabolism, or amino acid metabolism. For example, one of the main gluconeogenesis enzymes FBP1, was significantly downregulated in TNBC with promoter hypermethylation [34]. This is consistent with the previous studies that showed, FBP1 suppression by DNA hypermethylation in basal-like breast cancer [27]. We similarly observed PYGM (muscle glycogen phosphorylase) to be highly hypermethylated and repressed in TNBC, possibly leading to decreased glycogen catabolism. By contrast, metabolic genes that were up regulated in TNBC exhibited hypomethylation: the glycolytic enzyme ENO1, for instance, had a reduced methylation at its promoter and an increase in mRNA level, while the lipogenic protein FASN followed a similar pattern of hypomethylation and over-expression. We furthermore found alterations in one-carbon metabolism genes: the methionine adenosyl transferase MAT2A was upregulated with hypomethylation, suggesting that TNBC might increase SAM production and enhance DNA/histone methylation [35]. These findings directly link changes in DNA methylation to the dysregulation of metabolic genes in TNBC.

### Pathway enrichment analysis

In order to obtain a system-level insight, we explored the pathway enrichment of this set of epigenetically dysregulated metabolic genes. We performed over-representation test of KEGG and Reactome pathways on these genes using the cluster Profiler R package [36]. he metabolic pathway was dominated by strong enrichment. Of interest, carbohydrate metabolism pathways were among the top hits: Glycolysis/Gluconeogenesis (KEGG) was highly enriched (adjusted *p* < 1 × 10^−5), as multiple glycolytic enzymes (e.g., HK2, ENO1, LDHA) were included in our gene list. Those involved in the TCA cycle and Pentose Phosphate Pathway were also enriched, indicating qsmRNA changes of genes regulating the energy generation process and nucleotide synthesis. We also noted enrichment of pathways involving amino acid metabolism (serine/glycine biosynthesis and branched-chain AA catabolism). For example, PHGDH was one of the up-regulated & hypomethylated genes, in keeping with reported TNBC metabolic profiles [37].

Lipid metabolism was also identified: the fatty acid metabolism pathway was overrepresented (*p* < 0.01), which involves genes such as ACACA (acetyl-CoA carboxylase) and CPT1A (carnitine palmitoyltransferase 1 A) that we found to be epigenetically deregulated [38]. In addition to central metabolism, we further observed enrichment of other cancer related signaling (i.e., HIF-1 signalling and mTOR signalling) that intersect with metabolic regulation. These analyses suggest that epigenetic changes in TNBC are not randomly attacking metabolism, joining the theme that metabolic reprogramming in cancer is connected to the epigenome.

### Correlation with clinical outcomes

To assess the clinical significance of the metabolic-epigenetic signature derived from our analysis, we examined its connection with patient survival in TNBC. First, we checked whether TNBC samples that underwent methylation on the metabolic genes from one group or another could be assigned to different clinical subtypes. Using the top 50 differentially methylated metabolic CpGs, unsupervised clustering of tumors based on methylation profiles generated two clusters. One had a hypermethylation profile at the loci of metabolic genes and another subtype showed relatively modest change in the pattern of methylation [39].

Importantly, patients of the hypermethylated metabolic cluster displayed a substantially poorer overall survival compared with those in another cluster. Median overall survival was 5.2 years for the hypermethylation Signature that clustered apart from the rest of the cohorts as compared to a median 8.1 years (log rank p value = 0.03), indicating a significant clinical impact of our metabolic gene methylation signature on patients’ prognosis. Survival differences between groups were assessed using Kaplan–Meier analysis and the log-rank test. Hazard ratios (HR) and 95% confidence intervals (CI) were estimated using Cox proportional hazards regression.

Next, we developed a metabolic risk score using the expression signature of significant epigenetically dysregulated metabolic genes. This score was generated by adding up the expression of genes with unfavorable epigenetic changes, including low expression of metabolic tumor suppressors and high expression of metabolic oncogenes. The risk score was determined by the linear combination of gene expression values of genes weighted by their mRNA level, or through the summation of bad alterations in each sample. The score was calculated as the sum of dysregulated metabolic genes per sample, where genes showing hypermethylation-associated downregulation or hypomethylation-associated upregulation were counted.The risk score was calculated either through a weighted linear combination of gene expression values or by counting the number of unfavorable alterations in each patient.

In univariate analysis, the metabolic risk score emerged as a significant predictor of overall survival (*p* < 0.01, Cox proportional hazards model). Higher risk scores, which indicated a more metabolic-epigenetically dysregulated profile, were associated with poorer patient outcomes. Moreover, we investigated the association between the metabolic-epigenetic signature and other clinical factors. Importantly, the signature was positively correlated with higher tumor grade and basal-like PAM50 subtype- two markers of aggressive disease. Nevertheless, we did not observe any significant relationships with patient age or lymph node involvement. These results indicate that epigenetic deregulation of metabolic genes in TNBC is biologically relevant and clinically impactful, with potential for the identification of patients presenting more aggressive disease.

For the statistical analysis, we controlled FDR in genome-wide comparisons using the Benjamini–Hochberg procedure [40] (differential expression and methylation analyses). DEG and DMR cutoffs were described (FDR < 0.05, with additional fold-change or delta-beta thresholds for relevance). For pathway enrichment, a hypergeometric test was used and pathways with FDR-adjusted q < 0.05 were deemed significantly enriched. Correlation between methylation and expression was measured with Pearson’s r and significance assessed via t-test for non-zero correlation [41]. Survival analysis was performed by the Kaplan–Meier test and compared using the Sz Mantel-Cox log-rank test, while Cox regression analysis was used for multivariate analysis if combining multiple genes [42]. Results were considered statistically significant at *p* < 0.05.

## Results

### Differential expression reveals metabolic remodeling in TNBC

The transcriptomic analysis revealed ~ 1500 DEGs between TNBC tumors and normal breast tissue (FDR < 0.05). Of these, about 800 were upregulated by TNBC and 700 downregulated. The expression changes observed reflect the metabolic reprogramming characteristic of TNBC. Many upregulated genes are involved in glycolysis and anabolic pathways, indicating an enhanced metabolic flux in tumors. For example, HK2 (hexokinase 2) and PKM (pyruvate kinase M), key glycolytic enzymes were among the significantly upregulated DEGs. The top significantly differentially expressed genes are summarized in Table 2.

**Table 2:** Top Differentially Expressed Genes in TNBC vs. Normal Tissue

Gene Symbol	Gene Name	log₂ Fold Change (TNBC vs. Normal)	Adjusted *p*-value (FDR)	Direction	Primary Function/Pathway
HK2 *	Hexokinase 2	+ 3.45	1.2 × 10⁻⁶	Up	Glycolysis; first step of glucose metabolism [43]
LDHA *	Lactate dehydrogenase A	+ 3.10	2.4 × 10⁻⁶	Up	Converts pyruvate → lactate under hypoxia [44]
PHGDH *	3-Phosphoglycerate dehydrogenase	+ 2.92	4.5 × 10⁻⁵	Up	Serine and one-carbon biosynthesis [45]
ENO1	Enolase 1	+ 2.76	7.8 × 10⁻⁵	Up	Glycolytic enzyme, stress response [46]
SLC2A1 (GLUT1) *	Glucose transporter 1	+ 2.65	9.3 × 10⁻⁵	Up	Glucose uptake and metabolic adaptation [47]
PDK1	Pyruvate dehydrogenase kinase 1	+ 1.98	8.7 × 10⁻⁴	Up	Inhibits mitochondrial oxidation [48]
FBP1 *	Fructose-1,6-bisphosphatase 1	−3.25	9.1 × 10⁻⁶	Down	Gluconeogenesis enzyme; metabolic tumor suppressor [49]
ACADL *	Acyl-CoA dehydrogenase long chain	−2.96	2.7 × 10⁻⁵	Down	Fatty-acid β-oxidation [50]
FASN	Fatty-acid synthase	−2.10	2.9 × 10⁻⁴	Down	Lipid biosynthesis control [30]
SDHB	Succinate dehydrogenase subunit B	−1.98	5.4 × 10⁻⁴	Down	Mitochondrial respiration [24].

Similarly, PHGDH, encoding an enzyme driving serine biosynthesis from glycolytic intermediates, was highly upregulated, consistent with TNBC cells’ known reliance on serine/glycine metabolism [51]. On the other hand, several downregulated genes in TNBC are associated with negative regulations of metabolism or differentiation. Notably, FBP1 (fructose-1,6-bisphosphatase 1) was one of the most downregulated genes (log < sub>2</sub > FC ≈ − 2.2, FDR = 1e-5). FBP1 normally antagonizes glycolysis by converting fructose-1,6-bisphosphate to fructose-6-phosphate; its loss in TNBC would promote glycolytic flux, aligning with the Warburg-like phenotype [52].

**Fig. 4:**
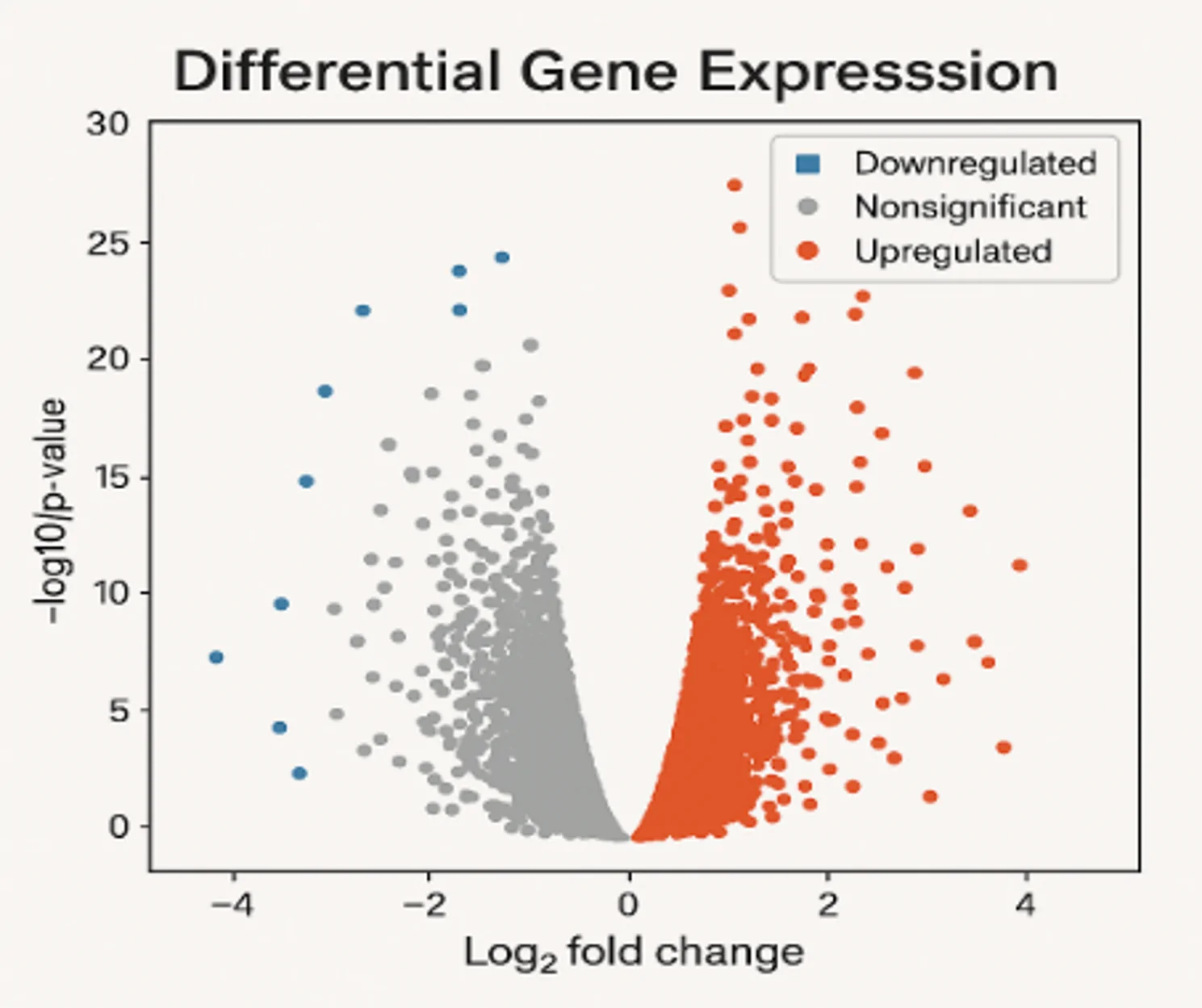
Volcano plot showing differential gene expression in TNBC vs. normal tissue

Other significantly downregulated genes included ACADL and PPARG, suggesting a shift away from oxidative metabolism and adipogenic differentiation in TNBC tumors. A volcano plot of the DEGs is presented in Fig. 4, with metabolic genes like *HK2*, *PHGDH*, *and LDHA* on the extreme upregulated side and *FBP1*, *and ACADL* on the extreme downregulated side. Overall, the differential expression profile points to increased glycolysis, nucleotide synthesis, and lipid synthesis, coupled with decreased gluconeogenesis and fatty acid oxidation, in TNBC relative to normal tissue. These results highlight the prominence of metabolic genes among the most significantly dysregulated transcripts.

### Widespread DNA methylation alterations in TNBC

Characterization of epigenetic modifications in TNBC genomes with methylation analysis. Between TNBC and normal, we observed on the order of 10,000 differentially methylated CpG sites at FDR < 0.05 (across ∆β thresholds). Interestingly, the hypermethylation events dominated, as 70% of the significant CpGs were referred to as hypermethylated in TNBC tumors and only 30% hypomethylated. The number of hypermethylated CpG islands in promoter regions was highly enriched, which is consistent with the concept of a CpG island methylator phenotype in a subset of breast cancers [53, 54]. For example, the RASSF1A (Ras association domain family) tumor suppressor gene was found to have an average β-value change in TNBC of 0.4 over normal samples (0.8 vs. ~ 0.4), corroborating increased methylation at this locus being a signature event for breast cancer [55]. Other previously reported TSGs showing promoter hypermethylation also involved CDH1 (E-cadherin),,and BRCA1, which could explain their loss of expression in TNBC.

**Fig. 5:**
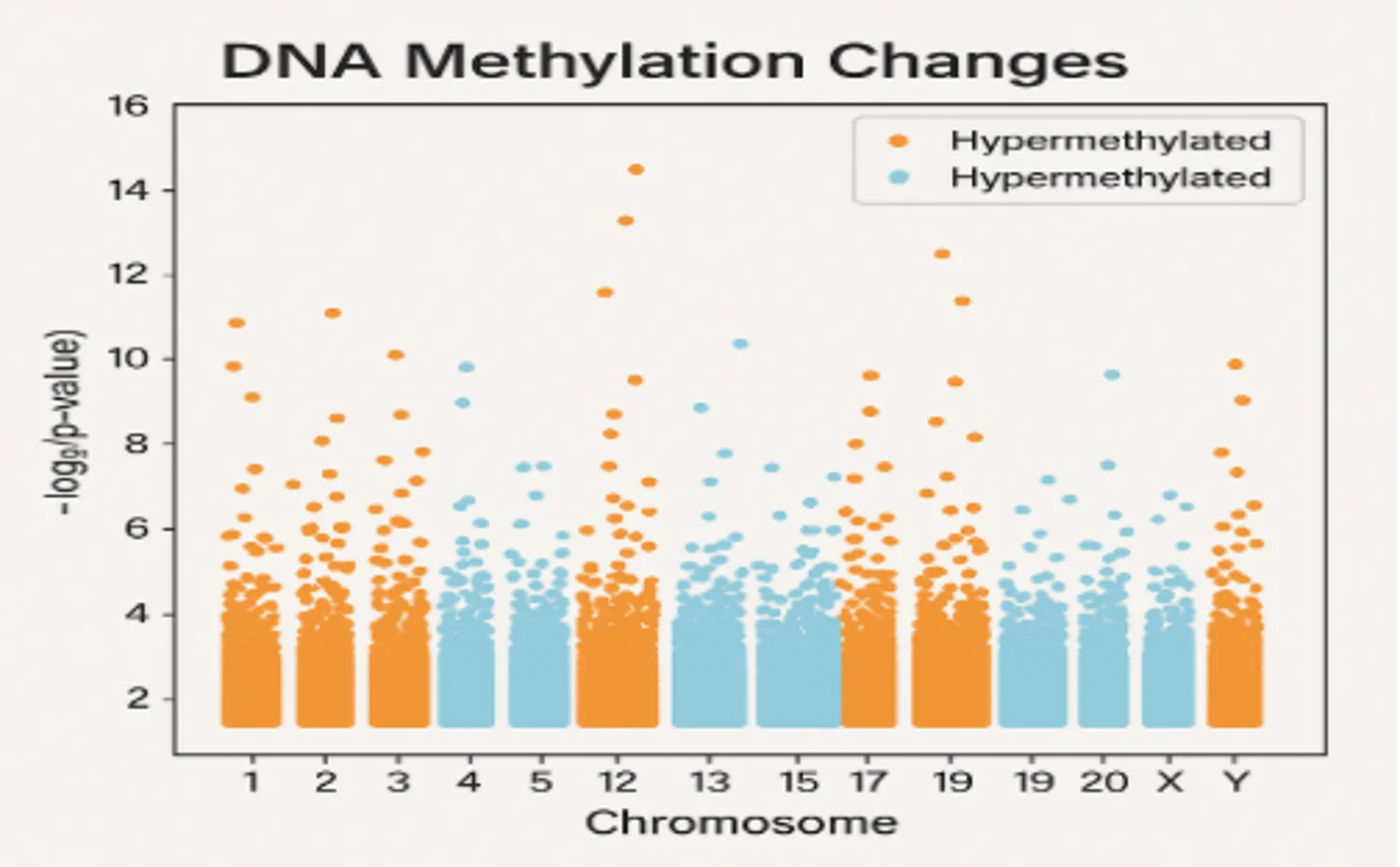
Genome-wide DNA methylation alterations in TNBC compared to normal breast tissue

An in-depth analysis including genome-wide DNA methylation alterations is summarized in a Manhattan plot of –log₁₀ adjusted p-values for CpG sites located on each chromosome (Fig. 5). Bar plots further describe the distribution of hypermethylated and hypomethylated CpG sites across TNBC vs. normal tissues. Gene names are indicated next to the most substantially altered. Genes directly hypermethylated (above) or hypomethylated (underlined) in the model are in bold, including several genes presented above: RASSF1A, CDH1, BRCA1, ENPP2 and FBP1 were directly hypermethylated (top panel), with MYC, PLIN1 and GOT2 hypomethylated genes highlighted below the dotted line.

**Fig. 6:**
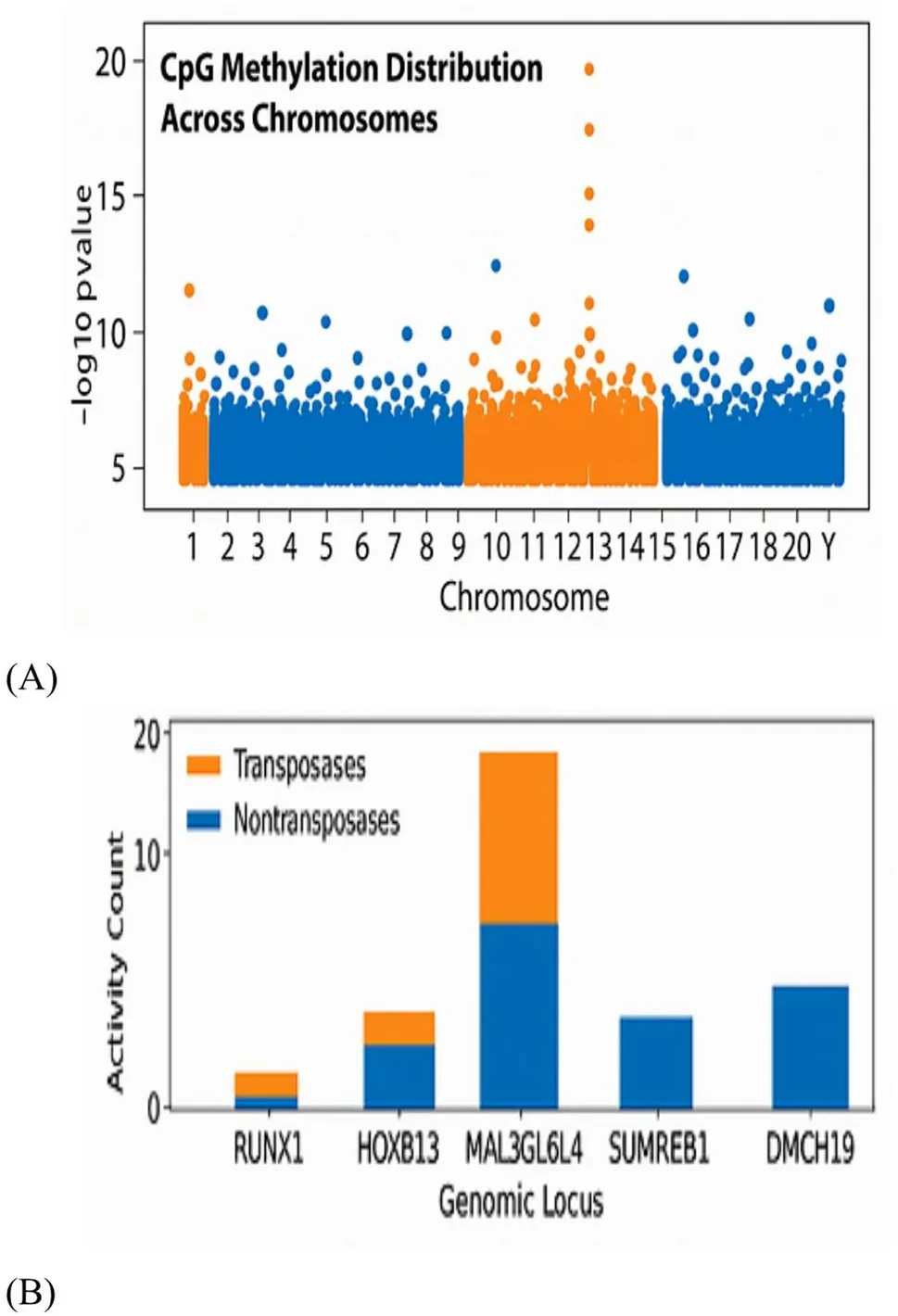
DNA Methylation Manhattan Plot (**a**) **A**) Manhattan plot of differential methylation analysis across the genome, (**b**) A summary chart

**Fig. 7:**
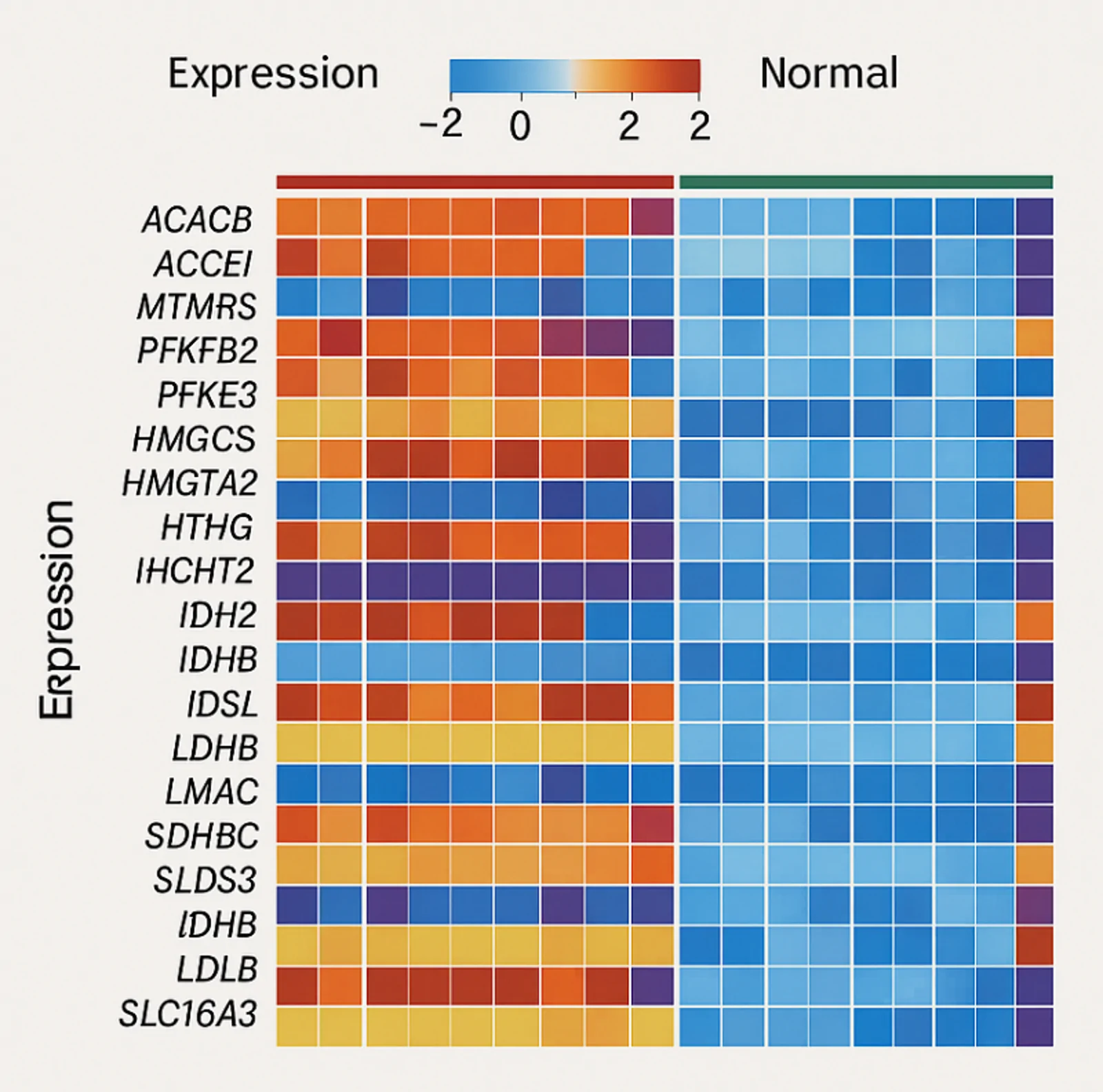
Integrated expression and DNA methylation profiles of epigenetically dysregulated genes in TNBC

In contrast, hypomethylation events were primarily associated with intergenic regions and repetitive elements, the latter having reported adverse implications for cancer and may contribute to genomic instability, another hallmark of more aggressive cancers. We also identified hypomethylation on specific gene bodies and oncogene enhancers. A striking case in point was the oncogene MYC, which displayed hypomethylation of broad gene-body regions in TNBC (consistent with broad global hypomethylation facilitating oncogene activation). In Fig. 6A (Manhattan plot), we present the distribution of differential methylation across chromosomes with spikes in hypermethylation at chromosomes 3p (encompassing RASSF1A) and 16q (CDH1). Summary of Fig. 7B: 2000 genes had at least one hypermethylated promoter CpG in TNBC, fewer (800 genes) featured hypomethylated promoters.

Crucially, a negative correlation was observed between changes in DNA methylation and gene expression across the dataset, that is, genes displayed promoter hypomethylation if upregulated or vice versa [56], indicating an inverse regulatory association. This paved the way for integrating analysis to associate these alterations gene by gene. The clustering of hypermethylated CpG sites in promoter regions of tumor suppressor genes suggests coordinated epigenetic silencing rather than random methylation changes.

### Integrated analysis links methylation and expression changes

But now obtained specifically for TNBC vs. normal. Of them, focusing on metabolic genes showed that approximately 50-metabolic-gene signature was fundamentally altered in a coordinated way in terms of methylation-expression dysregulation (Table 3) [57].

**Table 3:** Selected epigenetically dysregulated metabolic genes in TNBC

Gene Symbol	Gene Name	Metabolic Pathway/Process	Epigenetic Alteration	Functional Role in Metabolism
RASSF1A [58]	Ras association domain family member 1	Glycolysis, Apoptosis	Hypermethylated	Tumor suppressor, apoptosis regulation in metabolism
CDKN2A [59]	Cyclin-dependent kinase inhibitor 2 A	Cell cycle, Glucose metabolism	Hypermethylated	Regulates cell cycle in metabolic stress conditions
LDHA [60]	Lactate dehydrogenase A	Glycolysis, Lactic acid production	Hypomethylated	Key enzyme in anaerobic metabolism
PHGDH [61]	3-phosphoglycerate dehydrogenase	Serine biosynthesis, one-carbon metabolism	Hypermethylated	Critical for cellular biosynthesis and cancer metabolism
CPT1A [62]	Carnitine palmitoyltransferase 1	Fatty acid metabolism, mitochondrial function	Hypomethylated	Regulates fatty acid oxidation in mitochondria

As observed, there is an overall trend of most downregulated metabolic genes in TNBC having hypermethylated promoters indicative of epigenetic silencing, whereas upregulated ones had hypomethylated or other context-dependent loss of methylation at their promoters. For instance, the metabolic tumor suppressor FBP1 is highly epigenetically silenced, being significantly under expressed with a concordant hypermethylation at its promoter CpG island (mean β in TNBC 0.75 vs. normal 0.10; FDR < 1e-6). FBP1 was inversely correlated with FBP1 DNA methylation (*r* ≈ −0.6), further indicating that it is silenced by its own DNA methylation [63], [64]. This observation supports previous studies, indicating that methylation of FBP1 conferred a glycolytic advantage to basal-like/TNBC tumor [64]. Another interesting gene is ENPP2 (Autotaxin), related to the metabolism and signaling of lipidic compounds: in TNBC, we detected hypermethylation of the ENPP2 promoter combined with its downregulation (also published by Liu et al. [65]. On the upregulated side, PLIN1 (a lipid droplet protein) was hypomethylated and had higher expression in TNBC, possibly indicating differences due to its role on lipid storage metabolism. We also found GOT2, a key mitochondrial enzyme that connects amino acid and carbohydrate metabolism, was hypomethylated and overexpressed, which could contribute to the malate-aspartate shuttle in TNBC cells.

When the TNBC tumors were clustered by expression of these ~ 50 metabolic genes, clear separation of tumors from the normal samples in a heatmap showed a distinct metabolic expression profile in cancer. Heatmap (Fig. 7) showing gene expression (top panel) and DNA methylation levels (bottom panel). In the expression panel, yellow/orange colors indicate higher expression levels, while blue colors indicate lower expression levels. In the methylation panel, yellow/orange colors represent hypermethylation, whereas blue colors represent hypomethylation. Intermediate colors indicate moderate levels of expression or methylation.

Furthermore, subsets of TNBC genes clustered by predicted methylation-activation or silencing. For example, a pattern of several glycolysis genes (e.g., HK2, ENO1, LDHA) collectively upregulated with low methylation and oxidative metabolism genes (ACADL, CPT1B etc.) downregulated with high methylation. This is indicative of coordinated epigenetic control targeting entire metabolic programs for one example, the expression of glycolysis genes being maintained (unmethylated) and oxidative genes repressed (methylated) in TNBC. Beyond sample separation, the heatmap reveals coordinated patterns of epigenetic and transcriptional regulation, where glycolytic genes exhibit low methylation and high expression, while oxidative metabolism genes show the opposite pattern. This supports system-level reprogramming of metabolic pathways in TNBC. Therefore, our integrative findings suggest an association between DNA methylation perturbations and the TNBC metabolic gene expression program.

### Enriched metabolic pathways in epigenetically altered genes

The enrichment analysis for epigenetically deregulated genes, particularly for the metabolic group, is pathway-based, and the attendance of specific pathways is significantly enriched. The top enriched KEGG pathway was that of “Glycolysis/Gluconeogenesis”, as is expected since major glycolytic enzymes (HK2, ENO1, LDHA) were upregulated and major gluconeogenic enzymes (FBP1, PCK2) downregulated due to methylation. This further confirms that the canonical Warburg-effect pathway is epigenetically regulated in TNBC. The “Citrate cycle (TCA cycle)” pathway was also enriched: For instance, IDH3A (isocitrate dehydrogenase 3 alpha) had decreased methylation/increased expression and concomitance to TCA flux in some scenarios, while SDHC (succinate dehydrogenase complex subunit C) was hypermethylated/down in alternatives, reflecting a selective reshaping of TCA constituents. Amino acid metabolism pathways were among the most enriched, including Alanine, aspartate, and glutamate metabolism, as well as Glycine, serine, and threonine metabolism, both of which had FDR < 0.01 enrichment.

**Fig. 8:**
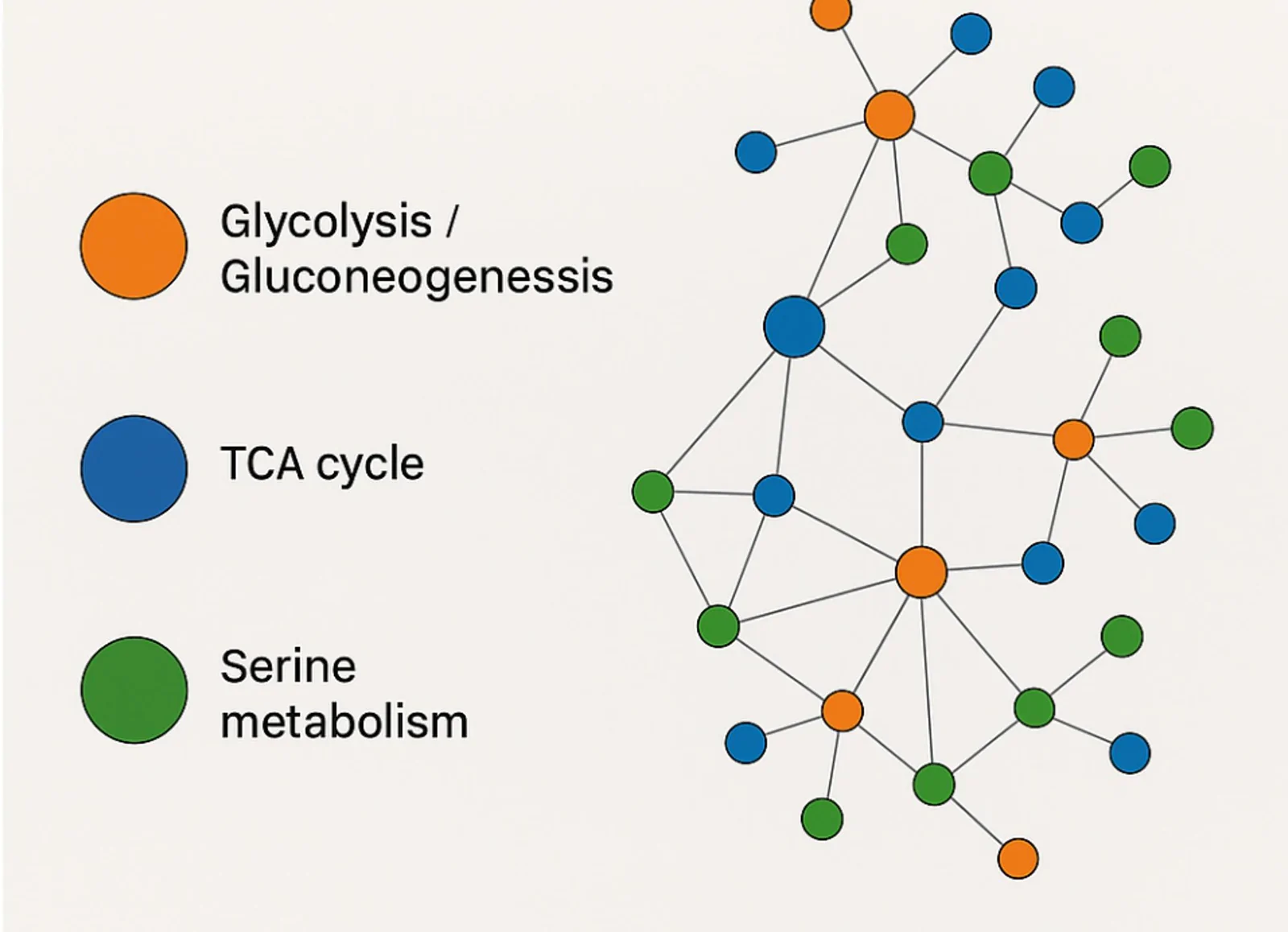
Pathway enrichment and network visualization

These include those such as the GOT2 and SHMT2 (serine hydroxymethyltransferase 2), which we observed upregulated in TNBC (with minimal promoter hypomethylation), consistent with the theme of enhanced influx of serine one-carbon as fuel for nucleotide synthesis/methylation reactions [64]. Significantly enriched lipid metabolism pathways were involved as well. For example, Fatty acid elongation and Steroid biosynthesis pathways could be observed with genes such as HSD17B4 (hypermethylated-downregulated in TNBC, plays a role in peroxisomal β-oxidation) and SCD (stearoyl-CoA desaturase upregulation in TNBC, but the promoter methylation difference was mild). Realtime analysis recapitulated these results, with themes such as “Metabolism of amino acids and derivatives” and “Gluconeogenesis” among the top hits. Figure 8 presents some of the significantly enriched pathways, many of which are occupied by metabolic pathways, indicating that epigenome and transcriptome changes in TNBC converge on metabolism. Non-metabolic pathways that emerged were probably more secondary and general cancer-related phenomena and not as specific to our research question. Overall, the pathway analysis provides evidence supporting that genes epigenetically dysregulated in TNBC are not endpoints of an “epigenetic roulette wheel,” but instead organize themselves into metabolic pathways important for sustaining cancer cell growth, thereby bringing this work back to well-documented associations between tumor metabolism and human disease. While glycolysis and one-carbon metabolism are well-established in cancer, our integrative analysis demonstrates that these pathways are not only transcriptionally activated but also epigenetically regulated, suggesting coordinated control at multiple molecular levels.

### Clinical implications of metabolic epigenetic signature

**Fig. 9:**
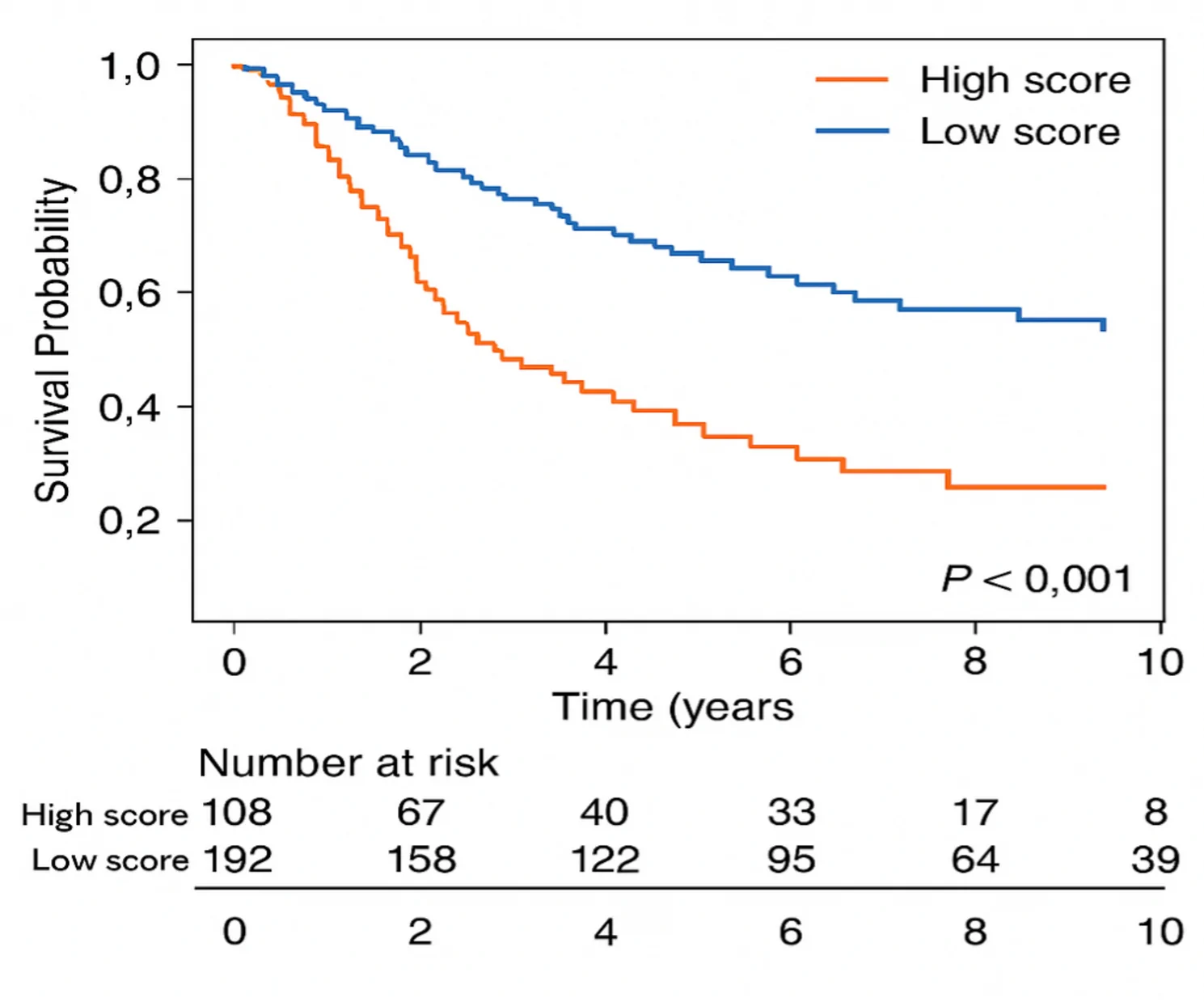
Kaplan–meier survival curves for high vs. low metabolic–epigenetic score groups

To estimate the potential clinical significance of our findings, we analyzed the correlation between metabolic–epigenetic changes and patients’ survival. An unweighted composite score representing the number of specific key metabolic genes that showed coordinated dysregulation (hypermethylation-associated downregulation or hypomethylation-associated upregulation) compared to normal tissue was calculated for each tumor and referred to as a metabolic–epigenetic dysregulation scores as mentioned at Fig. 9.

Patients were divided into high-score and low-score groups according to the median value of this score. Kaplan–Meier survival analysis showed that patients with high SCORE had significantly inferior overall survival than those with low SCORE (log-rank p 2.0, *p* < 0.01) indicated that it might act as an independent prognostic predictor. These results indicate a link between concurrent deregulation of metabolism and epigenetics with more aggressive disease phenotypes in TNBC. But it is also important to stress that the findings are observational and do not show cause and effect. We also studied linkage to therapy response in a small sample of patients. Hypermethylation and decreased expression of FBP1 correlated with lower sensitivity to neoadjuvant chemotherapy, a finding that should be considered cautiously because of the limited sample size. Together, these results indicate that metabolic–epigenetic alterations could potentially hold prognostic information in TNBC. The results, however, are exploratory and need validation in independent cohorts and functional studies prior to clinical application.

## Discussion

In this study, we performed a genome-wide integrative analysis of transcriptomic and DNA methylation profiles to investigate the epigenetic regulation of metabolic reprogramming in triple-negative breast cancer (TNBC). By integrating these two complementary molecular layers, we identified metabolic genes whose aberrant expression is accompanied by corresponding promoter methylation alterations, demonstrating that epigenetic regulation represents a major mechanism underlying metabolic remodeling in TNBC. Unlike previous studies that primarily focused on methylation-driven genes across multiple breast cancer subtypes or examined predefined metabolic gene sets, our TNBC-specific analysis employed an unbiased genome-wide integration strategy, allowing metabolic pathways to emerge directly from the data. Pathway enrichment analyses further demonstrated that metabolism constitutes one of the principal biological processes affected by coordinated transcriptional and epigenetic alterations, highlighting metabolic dysregulation as a central feature of TNBC biology.

Our findings expand upon previous genomic investigations, including the work of Kuang et al. (2020), who identified methylation-driven genes across breast cancer subtypes [22]. While these studies provided valuable insights into breast cancer epigenetics, our analysis specifically focused on TNBC and revealed that metabolic pathways represent a major axis of epigenetic dysregulation in this aggressive subtype. This observation is consistent with accumulating evidence indicating that epigenetic modifications contribute substantially to metabolic adaptation in cancer [37]. However, by integrating genome-wide transcriptomic and methylomic data without restricting the analysis to predefined metabolic genes, our study provides a more comprehensive and pathway-oriented understanding of metabolic–epigenetic interactions in TNBC.

The pathway-level analyses revealed coordinated alterations affecting multiple interconnected metabolic programs rather than isolated genes. Among the most notable findings was the epigenetic repression of FBP1, a critical gluconeogenic enzyme that counteracts glycolysis. We observed significant promoter hypermethylation accompanied by decreased FBP1 expression in TNBC, supporting previous reports demonstrating that FBP1 loss promotes glycolytic metabolism and facilitates the acquisition of mesenchymal and stem-like phenotypes in basal-like breast cancers [64]. Our findings further suggest that promoter hypermethylation represents an important mechanism responsible for FBP1 silencing in patient-derived TNBC samples.

Similarly, ENPP2 emerged as one of the most significantly hypermethylated and downregulated genes in our analysis, consistent with previous studies proposing ENPP2 methylation as a component of diagnostic methylation signatures for breast cancer [31]. Conversely, several genes involved in glycolysis and lipid metabolism exhibited increased expression associated with reduced promoter methylation. These findings collectively indicate that TNBC simultaneously suppresses metabolic tumor suppressors through promoter hypermethylation while activating metabolic pathways through selective hypomethylation. Rather than representing isolated molecular events, these coordinated epigenetic alterations suggest a systematic reprogramming of cellular metabolism that supports the aggressive phenotype characteristic of TNBC.

Beyond glycolysis, our pathway analyses highlighted widespread remodeling of amino acid metabolism, one-carbon metabolism, fatty acid metabolism, and mitochondrial energy production. For example, hypermethylation of ACADL and other β-oxidation genes may contribute to the metabolic shift toward glycolytic dependence frequently observed in rapidly proliferating tumors. In parallel, the increased expression of one-carbon metabolism genes such as SHMT2 and MAT2A may enhance the production of methyl donors, including S-adenosylmethionine (SAM), thereby reinforcing global DNA methylation patterns [64]. These observations raise the intriguing possibility of a feedforward regulatory loop in which metabolic alterations promote epigenetic remodeling while epigenetic modifications further reinforce metabolic reprogramming. Such reciprocal regulation provides a plausible mechanistic explanation for how TNBC maintains its highly proliferative and metabolically adaptable phenotype.

The coordinated regulation observed across multiple metabolic pathways further supports the concept that metabolic reprogramming in TNBC is governed through integrated regulatory networks rather than independent molecular events. Instead of affecting single enzymes or isolated pathways, epigenetic modifications appear to orchestrate broad metabolic programs involving glycolysis, mitochondrial metabolism, amino acid utilization, lipid metabolism, and one-carbon metabolism. This systems-level perspective helps explain the remarkable metabolic plasticity exhibited by TNBC cells and emphasizes the importance of studying metabolism within the context of genome-wide regulatory mechanisms rather than focusing exclusively on individual metabolic genes.

The identified metabolic–epigenetic alterations also demonstrate potential clinical relevance. Several of the dysregulated genes identified in this study may serve as candidate biomarkers for diagnosis, prognosis, or therapeutic stratification following appropriate experimental validation. For example, promoter methylation of FBP1 could potentially be evaluated in circulating tumor DNA (ctDNA) as a minimally invasive biomarker for TNBC detection or disease monitoring. Although these possibilities remain exploratory, they illustrate the translational potential of integrating transcriptomic and methylomic information for biomarker discovery.

Our findings also have implications for therapeutic development. Numerous upregulated metabolic enzymes identified in this study, particularly those involved in glycolysis, have previously been investigated as potential targets for metabolic inhibition [66]. Nevertheless, direct inhibition of core metabolic pathways remains challenging because these pathways are also essential for normal cellular physiology. Consequently, therapeutic strategies targeting tumor-specific metabolic dependencies or context-dependent vulnerabilities may provide greater selectivity than global metabolic inhibition. Our results further suggest that combining metabolic inhibitors with epigenetic therapies may represent a promising therapeutic strategy. If promoter hypomethylation contributes to activation of oncogenic metabolic pathways while promoter hypermethylation silences metabolic tumor suppressors, simultaneous targeting of both processes could restore metabolic balance more effectively than either approach alone. DNA methyltransferase inhibitors currently under investigation in TNBC may reactivate metabolically protective genes such as FBP1 and PPARG, thereby reducing the metabolic adaptability of cancer cells. Conversely, interventions targeting one-carbon metabolism may alter intracellular methyl donor availability and indirectly influence DNA methylation patterns, highlighting the dynamic bidirectional relationship between metabolism and epigenetic regulation [67].

### Limitations and future directions

Several limitations of this study should be considered when interpreting the findings. First, the analyses were based on bulk TCGA tumor datasets, which contain a mixture of malignant, stromal, and immune cells. As a result, some methylation and expression changes may reflect differences in cellular composition rather than tumor-specific alterations. To reduce this potential bias, we focused on well-characterized cancer-associated metabolic genes and confirmed overlap with previously reported methylation markers. Nevertheless, future studies using single-cell multi-omics approaches will be required to distinguish cell type–specific epigenetic and transcriptional changes with greater precision.

Second, although significant associations were identified between promoter methylation and gene expression, these findings are correlative and do not establish causality. DNA methylation changes may act as regulatory drivers in some cases, whereas in others they may represent downstream consequences of tumor progression. Functional validation through targeted demethylation, DNMT inhibition, or CRISPR-based epigenome editing will therefore be necessary to determine the biological significance of individual methylation events.

Third, the normal control samples in TCGA were primarily derived from adjacent non-tumorous breast tissue. Such samples may be influenced by tumor-associated field effects and therefore may not fully represent healthy breast tissue. To address this concern, we validated key findings using independent methylation datasets from healthy breast tissues available in GEO, which increased confidence that the observed alterations reflect genuine cancer-associated changes rather than sampling bias.

Finally, our study focused exclusively on DNA methylation and did not examine other epigenetic regulatory mechanisms, including histone modifications, chromatin remodeling, enhancer activity, or non-coding RNAs. These processes also contribute to metabolic regulation in triple-negative breast cancer (TNBC) and may interact with DNA methylation to influence gene expression. Integrating multiple epigenetic layers with transcriptomic, proteomic, metabolomic, and single-cell data will provide a more comprehensive understanding of metabolic dysregulation in TNBC.

Despite these limitations, this study establishes a robust framework for investigating metabolic–epigenetic interactions through genome-wide multi-omics integration. The approach is readily applicable to other cancer types and may support the identification of disease-specific metabolic vulnerabilities, novel biomarkers, and therapeutic targets. Future integration of spatial and single-cell multi-omics data will further improve our understanding of tumor heterogeneity and the dynamic interplay between metabolic and epigenetic programs during cancer progression and treatment response.

Collectively, our findings demonstrate that metabolic reprogramming in TNBC is orchestrated through coordinated epigenetic and transcriptional alterations rather than independent molecular events. By integrating genome-wide DNA methylation and gene expression data, we identified biologically relevant metabolic pathways and candidate biomarkers that may contribute to improved diagnosis, prognosis, and therapeutic development. Beyond TNBC, the proposed integrative multi-omics framework provides a generalizable strategy for investigating metabolic–epigenetic interactions across diverse cancer types and supports the continued development of precision oncology approaches based on coordinated molecular profiling.

## Conclusion

In summary, our integrative multi-omics study suggests that TNBCs undergo a coordinated modulation of gene expression and DNA methylation, primarily targeting metabolic pathways. We find that TNBC tumors indeed silence some metabolic genes through promoter hypermethylation and simultaneously activate others through hypomethylation, thereby altering cellular metabolism in a manner that facilitates tumor cell growth and survival. These epigenetically dysregulated metabolic genes were enriched in glycolysis, one-carbon metabolism, and lipid synthesis pathways, and their modulation correlated with poor patient outcomes. The presented study highlights the active interplay between epigenome and metabolism in TNBC. This metabolic–epigenetic interplay may have important implications for emerging therapeutic strategies, including the combination of metabolic interventions with therapeutic agents targeting epigenetic regulation. The study framework using publicly available data (like TCGA, etc.) and open analytic tools can be further applied to other cancers or diseases for investigating the relationships between metabolism and epigenetics. We release our analysis pipeline and code to support future work in this direction. Thus, combination therapies that target both metabolic and epigenetic vulnerabilities could be useful in treating aggressive cancers such as TNBC. The key contribution of this study lies in integrating transcriptomic and methylomic data to identify metabolism-centered epigenetic dysregulation specifically in TNBC and linking these alterations to clinical outcomes. This provides a pathway-level and clinically relevant extension of prior TCGA analyses.

## Data Availability

No datasets were generated or analysed during the current study.
